# Aqueous Rechargeable Zn/ZnO Battery Based on Deposition/Dissolution Chemistry

**DOI:** 10.3390/molecules27248664

**Published:** 2022-12-07

**Authors:** Vaiyapuri Soundharrajan, Jun Lee, Seokhun Kim, Dimas Yunianto Putro, Seulgi Lee, Balaji Sambandam, Vinod Mathew, Kumaresan Sakthiabirami, Jang-Yeon Hwang, Jaekook Kim

**Affiliations:** 1 Department of Materials Science and Engineering, Chonnam National University, Gwangju 61186, Republic of Korea; 2 Department of Prosthodontics, Dental Science Research Institute, School of Dentistry, Chonnam National University, Gwangju 61186, Republic of Korea

**Keywords:** aqueous rechargeable batteries, energy storage system, ZnO

## Abstract

Recently, a novel electrochemical regulation associated with a deposition/dissolution reaction on an electrode surface has been proven to show superiority in large-scale energy storage systems (ESSs). Hence, in the search for high-performance electrodes showcasing these novel regulations, we utilized a low-cost ZnO microsphere electrode to construct aqueous rechargeable batteries (ARBs) that supplied a harvestable and storable charge through electrochemical deposition/dissolution via a reversible manganese oxidation reaction (MOR)/manganese reduction reaction (MRR), respectively, induced by the inherent formation/dissolution of zinc basic sulfate in a mild aqueous electrolyte solution containing 2 M ZnSO_4_ and 0.2 M MnSO_4_.

## 1. Introduction

The development of smart and highly efficient energy storage systems (ESSs) is urgently required to enable practical grid-scale energy storage and to compensate for recent energy shortages [1]. Although lithium-ion batteries (LIBs) have dominated the ESS market in the past few decades, the persisting safety concerns of LIBs have motivated researchers to explore innocuous, environmentally friendly aqueous rechargeable batteries (ARBs) as the core components of next-generation ESSs [2,3,4,5]. Several ARBs exhibiting typical intercalation/deintercalation chemistries have been researched using various charge carriers, which include Li^+^, Na^+^, K^+^, Mg^2+^, Al^3+^, and Zn^2+^ [6,7,8,9,10,11], in the aqueous electrolyte solutions of their corresponding salts. Among these, aqueous rechargeable Zn-ion batteries (ZIBs), wherein Zn^2+^ functions as the charge carrier in a mild aqueous electrolyte, have recently emerged as one of the most promising battery technologies for ESSs. The outstanding feature of ZIBs is that metallic zinc can be directly used as an anode material with a high capacity (≈820 mAh g^−1^) [12], low working voltage (−0.762 V vs. a standard hydrogen electrode (SHE)), and remarkable energy density associated with the divalent ion (5851 mAh mL^−1^) [13]. However, researchers have struggled to identify an effective combination of potential Zn^2+^ storage hubs and tolerable electrolytes for ZIBs due to the dismal electrochemical reversibility and cycling stabilities of the main electrodes, mostly based on manganese oxide and vanadium oxide, respectively [14,15]. Interestingly, two recent studies suggested a reversible MnO_2_ deposition/dissolution reaction occurring on the electrode surface due to the reaction between the Mn^2+^ additive ions and water or ZBS, respectively, in the acidic electrolyte being the dominant contributor to the specific capacity in the corresponding manganese-cathode-constructed ARBs [16,17]. This mechanism can be facilitated in the presence of manganese, an element that is abundant in nature; can exist in various oxidation states (e.g., Mn^0^, Mn^2+^, Mn^3+^, Mn^4+^, and Mn^7+^) [18]; and, more importantly, can reversibly transition between water-soluble Mn^2+^ and solid MnO_2_. This reaction is analogous to the electrochemical manganese oxidation/reduction reactions (MORs/MRRs) suggested previously, and it is as follows [19,20]:

Mn^2+^ + 2H_2_O ↔ MnO_2_ + 4H^+^ + 2e^−^. (1)

However, these reactions have remained largely unexplored due to the lack of adequate materials showing compatible physical and electronic structures. Hence, they present challenging opportunities to explore inexpensive and common electrode materials that can effectively support the facilitation of highly reversible MORs/MRRs which are useful for energy storage device applications [21]. Perret et al. utilized graphene to support the reversible MnO_2_ deposition/dissolution in a hybrid supercapacitor, which demonstrated high energy and power densities of 25 W h kg^−1^ and 980 W kg^−1^, respectively, for at least 5000 cycles [22]. Surprisingly, recently developed aqueous manganese–hydrogen batteries exhibited a reversible Mn^2+^/MnO_2_ conversion on the cathode (porous carbon felt) surface and a H^+^/H_2_ conversion on the anode (Pt/C-catalyst-coated carbon felt) surface, delivering a high voltage (1.3 V), high energy density (≈139 W h kg^−1^), and good stability even after 10,000 cycles in the electrolyte (4 M MnSO_4_) [23]. Similarly, aqueous sodium–manganese batteries fabricated using a graphite-felt cathode, an activated-carbon anode, and a hybrid electrolyte consisting of 1 M Na_2_SO_4_, 1 M MnSO_4_, and 0.1 M H_2_SO_4_ also showed a reversible Mn^2+^/MnO_2_ conversion and exhibited a high voltage (1.2 V) and good stability over 7000 cycles [24]. Compared with graphite mats, metal oxides (MOs) are extensively available and environmentally benign and show fabrication scalabilities, admirable electrical conductivities, and good cycling stabilities [25,26,27,28]. More importantly, the wide range of multifunctional MOs offers numerous opportunities to develop high-performance energy storage devices through reversible Mn^2+^/MnO_2_ deposition/dissolution [28]. Furthermore, the use of a high-capacity/high-volumetric-energy-density metallic zinc anode and a mild aqueous acidic electrolyte (2 M ZnSO_4_ + 0.2 M MnSO_4_) mitigates the acidic-electrolyte-induced corrosion commonly observed in conventional energy storage devices.

In this paper, we identified the capability of ZnO in promoting highly reversible manganese deposition and dissolution efficiencies, good cycling stability, and a higher capacity. More specifically, ZnO showed the intriguing property of playing a sacrificial role via an electrochemically induced irreversible transformation into a layered zinc basic sulfate (ZBS) phase in the initial cycling, which, in turn, facilitated a highly efficient manganese deposition/dissolution process via a reversible MOR/MRR in a mildly acidic sulfate-based electrolyte solution.

## 2. Results and Discussion

**Figure 1:**
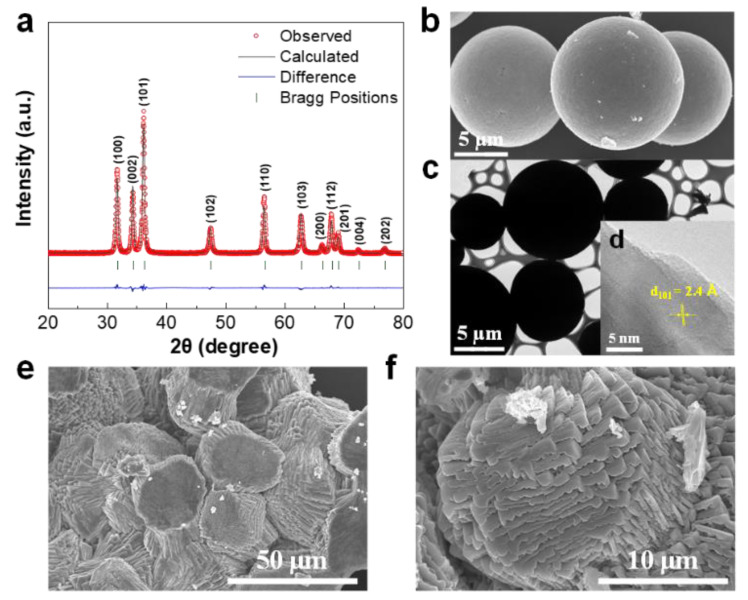
(**a**) XRD pattern of the as-synthesized ZnO microspheres, (**b**) FE-SEM image of ZnO microspheres, and (**c**) FE-TEM image of ZnO microspheres. Inset: (**d**) high-resolution TEM image of ZnO microspheres. FE-SEM image of ZnO microspheres treated with a ZMS solution for 1 h at (**e**) low and (**f**) high magnifications.

A uniform microspherical morphology of a ZnO electrode was prepared simply through a solvothermal reaction [29]. The detailed preparation procedure for the ZnO-microsphere is provided in the Experimental section. Rietveld refinement (Figure 1a) was conducted using FullProf software, and the obtained outputs suggested that the ZnO microspheres were indexed to a well-crystallized hexagonal structure. The lattice parameters obtained from the fitting of the ZnO microspheres are summarized in Table S1. Furthermore, the high-resolution (HR) FE-SEM image in Figure 1b showed that the produced ZnO microspheres exhibited an approximately 10–15 µm spherical core. The low-resolution TEM image in Figure 1c confirmed the nearly uniform distribution of the microspheres. The corresponding HR-TEM image (Figure 1d) revealed an interplanar distance of 0.24 nm, which agreed well with the characteristic (101) plane of the hexagonal ZnO identified from the XRD pattern. We found that ZnO exhibited, both chemically and electrochemically, an intriguing conversion property when exposed to a mild aqueous sulfate solution. To characterize this unique behavior of ZnO, we performed chemical and electrochemical studies in a mild aqueous sulfate solution which were performed as follows: Initially, SEM, XRD, XPS, and FTIR analyses were performed to interpret the intriguing feature of ZnO, i.e., the steady phase transition of ZnO into ZBS in the presence of mildly acidic sulfate-based aqueous solutions. The FE-SEM studies of ZnO immersed in a ZMS solution after 1 h are presented in Figure 1e,f. It is clear that the uniform ZnO microspheres transformed into hierarchical microspheres that were fully covered with a flake-like morphology. The chemical conversion of the ZnO microsphere into the ZBS phase in mildly acidic sulfate-based solutions (ZS and ZMS solutions after a 1 h dip) was verified through the XRD outputs in Figure S1.

To gain further insights into the changes in the surface chemistry after the sulfate-induced conversion, FTIR analysis was used to analyze pristine ZnO powder. Layered zinc sulfate hydroxide samples were formed in the two solutions of ZS and ZMS. The FTIR spectra of the pristine ZnO sample showed two dominant vibrations at 430 cm^−1^ and 520 cm^−1^ as presented in Figure S2a. These vibrational numbers were in agreement with the classical absorption features of Zn–O and Zn–OH, respectively [30]. Also, the same two fingerprints were observed with relatively fewer absorption intensities in the FTIR spectra of the ZnO samples treated with the ZS and ZMS solutions.

More importantly, in the range of 600–1200 cm^−1^, the typical vibrations of intercalated sulfate anions were observed in both samples [30]. Specifically, the sharp peak at 1119 cm^−1^ represented SO_4_^2−^ group stretching vibrations, and the weak band at ≈611 cm^−1^ was attributable to the O–S–O bending mode [31]. Broadbands appearing in the high-frequency range of 3500–3700 cm^−1^ were attributed to the OH group stretching vibrations of free water molecules, suggesting that the hydroxyl groups were occupied with hydrogen bonding and that the H_2_O molecules were in the interstitial layers [32]. Moreover, the band evolved at 1660 cm^−1^ was attributed to the H–O–H bending of water molecules in the ZBS interlayer [33]. The FTIR results, thus, verified the conversion of partial ZnO into ZBS in the presence of sulfate-based aqueous solutions. To further elucidate the chemical compositions and elemental variations, high-resolution Zn 2p and S 2p XPS spectra were measured for the pristine ZnO and the ZnO treated with sulfate-based ZS and ZMS aqueous solutions, respectively. The recorded profiles are presented in Figure S2b and clearly demonstrated that the Zn 2p3/2 peak slightly shifted toward higher binding energies when pristine ZnO powder (1021.22 eV) was immersed in ZS (1022.28 eV) and ZMS (1022.35 eV) solutions [34]. This suggested that ZnO showed a small chemical variation in the Zn_4_(OH)_6_SO_4_·xH_2_O content during the conversion. Similarly, S 2p footprints were absent in the pristine ZnO (Figure S2c), whereas their presence was confirmed in the ZS- and ZMS-treated ZnO samples, confirming the formation of Zn_4_(OH)_6_SO_4_·xH_2_O/ZnO in the conversion products. Moreover, the survey spectrum that was provided for all three samples (see Figure S3) further validated the formation of multiple elemental features during conversion. Overall, we demonstrated that the chemical reaction of ZnO powders in sulfate-based solutions was described by the gradual chemical transformation of ZnO into a ZBS/ZnO composite, which was induced by the acidic environment (pH) of the solution.

**Figure 2:**
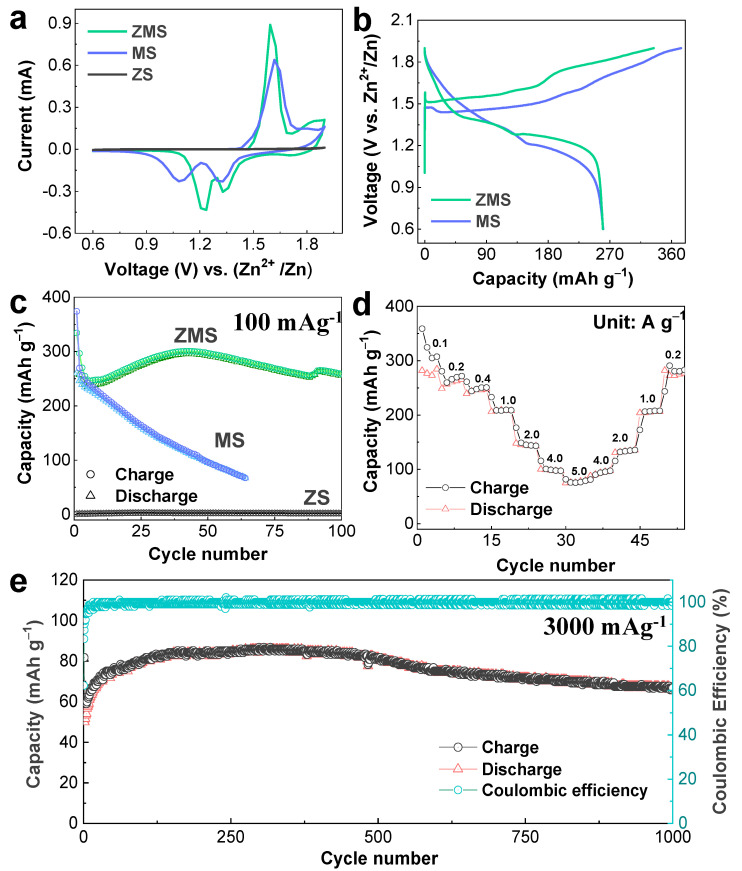
(**a**) CV curve of ZnO microspheres at 0.1 mV s^−1^ rate in ZS, MS, and ZMS electrolytes, (**b**) charge/discharge patterns of ZnO microspheres at 100 mA g^−1^ in MS and ZMS electrolytes for the first cycle, and (**c**) the corresponding cyclability profiles of the different electrolytes. (**d**) The rate capability profile of ZnO microspheres at various current rates in the ZMS electrolyte and (**e**) cyclability profile of ZnO microspheres at a current density of 3000 mA g^−1^ in the ZMS electrolyte.

These observations validated the unique gradual transition of ZnO into ZBS phases in mildly acidic sulfate-based solutions over a period of time. Thus, the unique phase transition characteristics during the chemical reaction of ZnO with sulfate-based aqueous solutions motivated the study of the electrochemical reactivity of ZnO under similar conditions that utilize acidic electrolytes. Simply said, upon the utilization of ZnO as an electrode in the mildly acidic sulfate-based electrolytes of ARBs, the phase transition characteristics of ZnO during the electrochemical reaction required monitoring. In fact, it can be expected that the possible electrochemical phase transformation of ZnO could hugely influence the reaction mechanism associated with reversible heterogeneous electrochemical MnO_2_ deposition/dissolution via a MOR/MRR. To realize this, we utilized ZnO as an electrode to study the electrochemical reaction of ZnO in the mildly acidic sulfate-based electrolytes of a typical ARB. Figure 2a depicts the initial CV patterns obtained for the ZnO-microsphere cathode cycled in 2 M ZnSO_4_ and in 0.2 M MnSO_4_ (ZMS), 0.2 M MnSO_4_ (MS), and 2 M ZnSO_4_ (ZS) electrolytes. Interestingly, the ZnO electrodes in ZMS and MS electrolytes produced identical CV patterns. Specifically, the Mn^2+^ → Mn^4+^ oxidation at 1.65 V and the successive Mn^4+^ → Mn^2+^ reductions at 1.3 V and 1.2 V were shown. Thus, it could be observed that the ZnO microspheres could reversibly deposit/dissolve MnO_2_ from/into the Mn^2+^ ions from the MnSO_4_ acting as a full-time electrolyte or as an Mn^2+^ donor. Intriguingly, earlier studies claimed that the peak at 1.3 V was due to the participation of H^+^ ions in the reduction of MnO_2_ into MnOOH in the Zn/MnO_2_ device following the deposition/dissolution reactions [28]. Conversely, ZnO revealed its idleness in the absence of Mn^2+^ ions because no oxidation/reduction peaks could be observed in the corresponding CV curve (Figure 2a). Furthermore, galvanostatic measurements were conducted for the ZnO-microsphere cathode cycled in MS and ZMS electrolytes at 100 mA g^−1^, and the resulting charge/discharge curves are shown in Figure 2b. Noticeably, manganese was deposited from/dissolved into both the MS and ZMS electrolytes with little voltage polarization. Specifically, two distinct charging plateaus centered at 1.6 V and 1.5 V (corresponding to the ZMS and MS electrolytes, respectively) indicated oxidation.

Similarly, the discharge curve exhibited two distinct reduction plateaus at the average potentials of 1.35 V and 1.2 V in the ZMS and MS electrolytes, respectively. The charge/discharge curves exhibited voltage polarization; however, the curve shapes and supplied discharge capacities (≈260 mAh g^−1^) were almost identical in both electrolytes. Figure 2c shows the cyclability curves of the electrodes cycled in ZMS, MS, and ZS at an applied density of 100 mA g^−1^. Although the initial discharge capacities of Zn/ZnO-microsphere-based cells employing ZMS and MS electrolytes exhibited identical discharge capacities (260 mAh g^−1^), the ZnO-cathode capacity in the latter decreased drastically to 67 mAh g^−1^ after the 64th cycle.

The ZnO-microsphere cathode was activated in the ZMS electrolyte solution owing to repeated ZBS formation and dissolution, which ensured the highly efficient reversible MORs/MRRs that caused the capacity to increase with an increasing number of cycles. The maximum activation capacity reached 296 mAh g^−1^ after the 43rd cycle. After a steady state was reached, the ZnO-cathode capacity slightly decreased, delivering a reversible capacity of 255 mAh g^−1^ after 100 cycles in the ZMS electrolyte solution. Importantly, the constructed Zn/ZnO energy device delivered a very high specific energy of 344 W h kg^−1^, even after 100 cycles, owing to highly reversible manganese deposition and dissolution on the ZnO cathode. In contrast, the absence of ZnSO_4_ in the MS electrolyte tended to minimize ZBS formation, which was essential to enhance reversible MnO_2_ deposition/dissolution. This ultimately led to decreased electrochemical reactivity and, hence, the mediocre performance of the ZnO-microsphere cathode throughout repeated cycling in the MS electrolyte (Figure 2c). Compared with the cathode performances of the ZnO microspheres in both the ZMS and MS electrolytes, a negligible capacity was measured in the presence of the ZS electrolyte, demonstrating that MORs/MRRs were not realized in the absence of Mn^2+^ ions in the electrolyte (Figure 2c). To further assess ZnO-cathode manganese deposition and dissolution processabilities under different current drains in ZMS electrolytes, we cycled the ZnO-microsphere cathode at alternating current rates, oscillating from 100 mA g^−1^ to 5000 mA g^−1^, and the resulting profiles are shown in Figure 2d. The reversible discharge capacities of the constructed Zn/ZnO battery in a ZMS electrolyte solution were 281, 256, 243, 207, 145, 99, and 76 mAh g^−1^ at 100, 200, 400, 1000, 2000, 4000, and 5000 mA g^−1^, respectively. Furthermore, when the reverse-current densities decreased, the capacity yields were identical to those of the previous current densities, signifying an excellent rate capability; thus, the ZnO cathode showed superior MORs and MRRs. Additionally, the corresponding charge/discharge curves used to determine the rate performance are included in Figure S4. The curves demonstrated similar voltage platforms at different current surges. As shown in Figure 2e, the ZnO microspheres maintained a high current density (3000 mAg^−1^) after 1000 cycles, and a reversible capacity of 68 mAh g^−1^ was measured at the 1000th cycle, which was still higher than the measured initial capacity (50.8 mAh g^−1^). This result demonstrated that the ZnO-microsphere electrode exhibited enhanced manganese-conversion reaction kinetics even at high applied current rates.

In the previous section, we demonstrated that the ZnO powder exhibited an interesting conversion behavior in sulfate-based solutions. We also performed a soaking experiment for the ZnO cathode at different reaction times (1 h, 24 h) in the ZMS electrolyte (Figure S5). From the soaking experiment, we witnessed a clear change on the surface of the ZnO electrode, where a thin white layer covered the surface of the cathode after 24 h of resting time (digital photograph of the inset in Figure S5). To verify the composition of the white layer, we performed an XRD analysis. From the XRD outputs, we found that the ZnO electrode transformed into the ZBS phase after 1 h. Moreover, after 24 h of dipping, the hydration level of ZBS and the intensity of the ZBS phase also increased. Additionally, the pH of the electrolyte after 24 h of dipping increased from 4 to 4.3 due to the chemical conversion reaction. To analyze the structural behavior of the white layer formed on the surface of the cathode, we performed a SEM analysis (Figure S6a–d). It could be observed that the white particles on the surface of the ZnO electrode were composed of microflakes, which are typical structures for the ZBS. With an increase in the dipping time, the microflakes entirely covered the surface of the electrode, and the results confirmed the proposed chemical conversion behavior of the ZnO electrodes when subjected to sulfate-based electrolytes.

**Figure 3:**
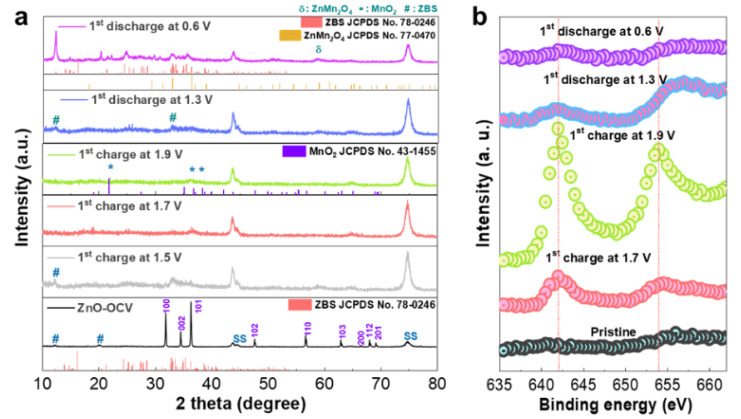
(**a**) Ex situ XRD analysis at different depths of charge/discharge voltages in ZMS electrolyte and (**b**) ex situ XPS profiles of Mn 2p at different depths of charge/discharge voltages in ZMS electrolyte.

To elucidate the electrochemical reaction of the ZnO-microsphere cathode and, hence, the energy storage mechanism behind the constructed Zn/ZnO battery, the electrodes were analyzed at different states of charge (SOC) and depths of discharge (DOD) using ex situ XRD for the initial electrochemical cycling in ZMS, and the resulting patterns are shown in Figure 3a. For a comparative assessment, the XRD patterns of the electrode before cycling were also included. The electrode recovered after a few minutes of contact with the electrolyte in the test cell and was washed/dried for structural measurements. The resultant XRD profile obtained for ZMS (Figure 3a) showed that, in addition to the ZnO phase, a new set of peaks at 2θ = 12° and 22° corresponding to chemical ZBS precipitation emerged. This confirmed our earlier observation from the study on the chemical reaction of ZnO immersed in the same mildly acidic ZMS solution. However, upon the initial charge of the ZnO electrode to 1.5 V SOC, the peak intensities of the crystalline ZnO noticeably diminished, while new broad peaks (around scanning angles 2θ = 33–36°) corresponded to the ZBS emergence. At a higher initial charging voltage of 1.7 V SOC, both the ZnO and ZBS peaks disappeared. At the cut-off charging potential (≈1.9 V SOC), small broadened traces corresponding to MnO_2_ (JCPDS no. 43-1455) emerged (Figure 3a). The following conclusions could be made based on these observations: (1) The large reduction in the characteristic peak intensities of ZnO observed with increasing SOC in the initial charge cycle suggested the transformation of crystalline ZnO. (2) The evolution of characteristic ZBS peaks into a pure phase during early charging suggested ZBS precipitation on the electrode surface during the electrochemical reaction. (3) At the application of an electrochemical charge potential, the facile and complete transformation of ZnO into the ZBS phase occurred compared with the ZnO/ZBS composite formation observed for the chemical reaction between the ZnO powders and aqueous sulfate solutions (Figure 1 and Figures S1–S3). The subsequent disappearance of the ZBS peaks followed by the broad features of MnO_2_ suggested the disintegration of ZBS. (4) ZBS disintegration induced heterogeneous MnO_2_ deposition on the cathode as observed by the emergence of phase-pure broadened MnO_2_ peaks upon complete charging. Although an earlier report proposed the feasibility of the parasitic ZBS phase heterogeneously reacting with the Mn^2+^ additive in the electrolyte to deposit MnO_2_ on the electrode surface [35], we believed that ZBS disintegration induced and supplied the energy for the heterogeneous electrodeposition of MnO_2_.

During discharge, ZBS footprints (JCPDS no. 78-0246) reappeared because intense peaks corresponding to the characteristics (111) and (100) planes were observed at scanning angles of 12° and 22°, respectively [36]. More importantly, an ex situ XRD analysis was performed for the ZnO electrode after the first charge and discharge process in the MS electrolyte. The ex situ XRD outputs of the MS electrolyte exhibited a close resemblance to that of the ZMS electrolyte (Figure S7), validating that the Mn^2+^ ions from the MS electrolyte were the key contributors in the proposed deposition/dissolution mechanism induced by the inherent formation/dissolution of zinc basic sulfate in a mild aqueous electrolyte solution containing MnSO_4_. This demonstrated the reversible formation of layered ZBS in the Zn/ZnO cell in both ZMS and MS electrolytes during the initial cycling. MnO_2_ with very low crystallinity was indeed deposited during charging, while ZBS formation dominated during discharge. Importantly, the dominant ZBS formation in the discharge reaction may prevent the characterization of a MnO_2_ dissolution reaction through ex situ XRD analysis. Thus, it is possible that the self-disintegration/regeneration of ZBS could facilitate MnO_2_ deposition/dissolution during the electrochemical reaction in the corresponding ARB.

To acquire evidence of the surface-based MnO_2_ dissolution reaction that was possibly masked by a dominant ZBS phase formation reaction during discharge, an XPS analysis of the cathodes was performed ex situ. Figure 3b depicts the ex situ Mn 2p XPS spectra of the cathode recovered at various initial SOC/DOC. Visibly, Mn 2p peaks started appearing during initial charging (≈1.7 V SOC), and, by the end of charging (≈1.9 V SOC), the Mn 2p peaks at 642.07 eV (2p3/2) and 653.9 eV (2p1/2) evolved considerably. In contrast, the XPS spectra of the pristine, nonreacted ZnO sample were devoid of Mn 2p peaks, indicating that manganese was deposited on the ZnO electrode surface during initial charging. The intensity of the peak attributed to Mn 2p, on the other hand, started reducing during discharge (≈1.3 V DOD) and was almost completely suppressed at the end of discharge (≈0.6 V DOD). However, the remnant Mn 2p perturbations that were distinguishable indicated that complete manganese dissolution was not achieved upon complete discharge.

**Figure 4:**
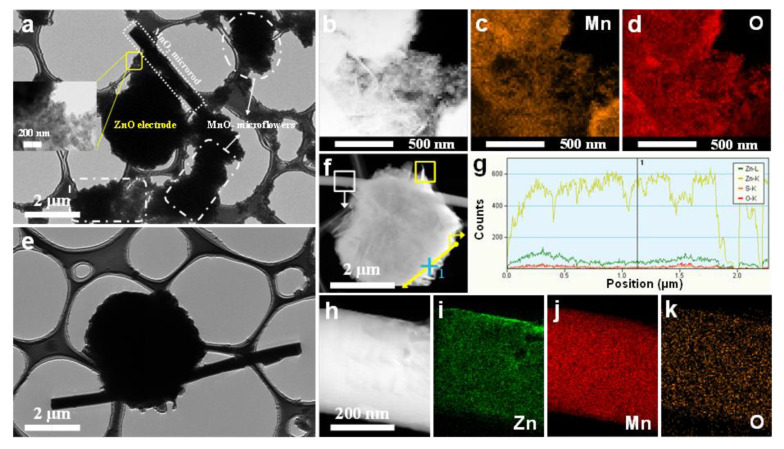
Ex situ TEM image of ZnO microspheres at 100 mA g^−1^ current density in ZMS electrolyte; (**a**) low-resolution image after 1st charge and high-resolution TEM image of the in-situ-formed microflowers (inset of a); (**b**–**d**) elemental mapping of the in-situ-formed microflowers in the charging process; (**e**) low-resolution image after 1st discharge, (**f**) brightfield image of the ZnO microspheres with the corresponding (**g**) elemental line profile of the nanoflakes on the ZnO microspheres Zn, S, and O; and (**h**–**k**) elemental mapping of the microrod after 1st discharge process.

The electrode sample was then examined with an ex situ TEM postmortem to determine its morphological evolution, and the results are presented in Figure 4. The cathode morphology after charging is depicted in Figure 4a and indicated that the original spherical shape was retained, sheltering microflowers and microrods (indicated by white dashed lines). The HR-TEM image of a microflower adhered to the ZnO microspheres is shown in the inset of Figure 4a. The selected-area elemental mapping analysis (Figure 4b) revealed that manganese and oxygen were uniformly distributed (Figure 4c,d, respectively), suggesting electrolytic manganese deposition. To obtain a detailed understanding of the electrolytic deposition of MnO_2_ during the charging process, high-resolution TEM targeted the observed microflowers. The corresponding interplanar spacing (0.32 nm, (201)) was close to the ramsdellite MnO_2_, and the imprecise SAED ring pattern of the microflowers verified that MnO_2_ was in the amorphous form on the surface of the ZnO cathode (Figure S8a–c). Moreover, the HR-TEM images of the charged product contained hierarchical microrods and microfibers as well. The lattice interplanar measurements (0.24 nm, (210)) and crystalline SAED pattern results ((210), (312), (121)) of the hierarchical microrods in Figure S8d–f indicated that electrolytic crystalline MnO_2_ was also deposited on the surface of the cathode. However, the amorphous lattice fringes and SAED pattern of the microfibers in Figure S8g–i could be due to the short fibers adhered to the cathode surface. In general, electrolytic MnO_2_ did not have any specific crystal phase. There was a combination of crystalline and amorphous MnO_2_, and our observation also confirmed the deposition of MnO_2_ in the crystalline and amorphous forms.

The morphology of the discharged ZnO electrode is illustrated in Figure 4e. The microflowers dissolved during discharge, indicating the dissolution of deposited manganese oxide. However, the ZnO microspheres were still surrounded by several nanoflakes, possibly owing to ZBS formation. Line mapping analyses of completely discharged products (≈0.6 V DOD) along the highlighted area in the brightfield image of the microflower in Figure 4f showed the distributed Zn, O, and S atoms, confirming ZBS formation (Figure 4g). More importantly, microrods formed during charging, and ZnO microspheres still coexisted, indicating the existence of short fibers from the separator, where it was highly possible for the growth of electrolytic MnO_2_ on the short fibers adhered to the face of the active material. Elemental mapping analyses of the microrods indicated that they were comprised of Zn, Mn, and O (Figure 4h–k, respectively), possibly owing to the electrochemical interaction of Zn^2+^ as either ZnMn_2_O_4_ or Zn_x_H_y_MnO_2_.nH_2_O into nondissolved MnO_2_ during discharge, which was consistent with previous reports [37,38].

Surprisingly, reversible manganese deposition and dissolution were identified inside the coil-cell setup via digital photography during ex situ postmortem analysis (Figure S9). After charging, a thin electrodeposited film adhered to the glass-fiber separator facing the electrode and adhered along the borders of the stainless-steel cap, suggesting the manganese deposition process (Figure S9a). Through SEM, we examined the electrodeposited film on the glass-fiber separator, where a thick layer covered the surface of the separator (microfibers), indicating the deposition of MnO_2_ (Figure S10a,b). The stainless-steel cap may have also served as a current collector, inducing manganese deposition. Instead, the absence of electrodeposited films on both the glass fibers and the stainless-steel cap in the pictures of the discharged cell components (Figure S9b) indicated that deposited manganese oxides dissolved back into the electrolyte. Overall, the detailed reaction mechanism of the Zn/ZnO battery fabricated using a manganese-sulfate-based electrolyte was analyzed through ex situ XPS, XRD, and TEM.

These results suggested that MORs and MRRs occurred during charging and discharge, respectively. In the following, we presented the distinctively fast electrochemical conversion of Mn^2+^ (liquid) to MnO_2_ (solid) (i.e., manganese deposition) during charging and the (quasi) reversible dissolution of deposited MnO_2_ as Mn^2+^ ions back into the electrolyte, which is a distinctive observation for a battery system constructed using manganese-based electrolytes. Thus, the results of the physical and chemical analyses suggested that the following ZMS chemical and electrochemical reactions described the Zn/ZnO-cell charge storage mechanism.

Cathode:

Before cycling:

ZnO + ZnSO_4_ + nH_2_O → Zn_4_SO_4_(OH)_6_∙xH_2_O (2)

Cycling:

2Zn_4_(OH)_6_SO_4_.nH_2_O + 3Mn^2+^ ↔ 3MnO_2_∙aH_2_O + 2SO_4_^2−^ + 8Zn^2+^ + bH_2_O + 6e^−^ (3)

4Zn^2+^ + SO_4_^2−^ + 6OH^−^+5H_2_O ↔ Zn_4_SO_4_(OH)_6_∙5H_2_O (4)

Anode:

Zn^2+^+2e^−^ ↔ Zn (5)

**Scheme 1:**
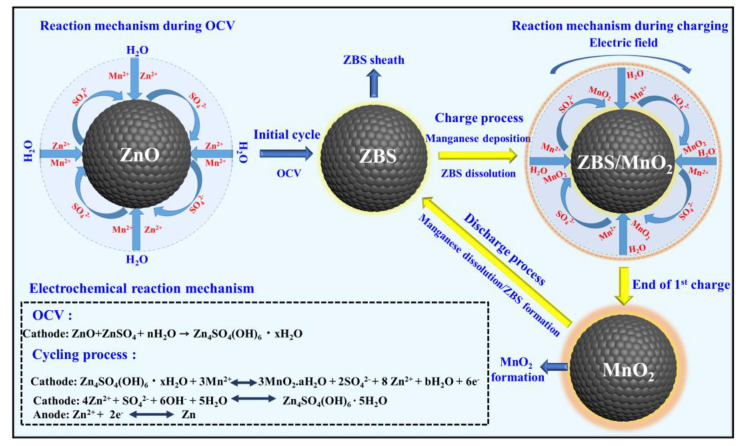
Electrochemical reaction mechanism inside the Zn/ZnO battery.

A schematic illustration of the reaction mechanism in the Zn/ZnO cell is provided in Scheme 1. The present study further demonstrated that the parental ZnO itself could be irreversibly transformed into ZBS, particularly by facile electrochemical reactions. Such a reaction induced heterogeneous MnO_2_ deposition/dissolution as described in the above equations [37].

Interestingly, the reversible MnO_2_-deposition/dissolution-induced charge storage mechanism was found to occur in diverse electrolyte solutions containing 1 M Na_2_SO_4_ or 1 M MgSO_4_ salts with a 0.2 M MnSO_4_ combination, respectively. The reversible manganese deposition/dissolution was associated with reversible ZBS formation/dissolution, demonstrating the diversity of Zn/ZnO cells containing sulfate-based electrolytes, a detailed discussion of which is provided in Supplementary Note 4 (Figures S11 and S12a).

**Figure 5:**
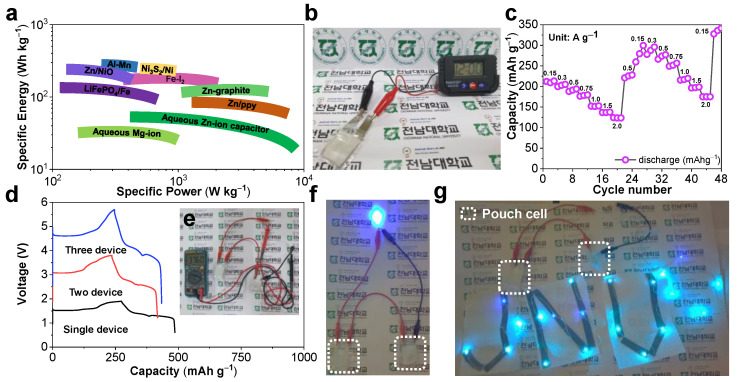
(**a**) Ragone plot of the Zn/ZnO battery compared with other reported battery devices, (**b**) selected charge/discharge patterns for the Zn/ZnO pouch battery cycled at different rates in ZMS electrolyte, (**c**) rate performance of the single-pouch Zn/ZnO battery, (**d**) galvanostatic charge/discharge profiles measured at a current density of 100 mA g^−1^ of a single-pouch battery and tandem devices with two- and three-battery units allied in series, (**e**) digital photograph representing the output voltage (3.7 V) of a two-pouch Zn/ZnO battery connected in series (after the 5th charge), (**f**) digital photograph of a single LED powered by a two-pouch Zn/ZnO battery connected in series, and (**g**) digital photograph of 1 m long serial LEDs powered by a two-pouch Zn/ZnO battery connected in series. (Explanation in Korean in the backround represents Chonnam National University).

For a practical evaluation, a Ragone plot that compared the energy and power density of the presently designed Zn/ZnO battery with other energy storage devices is depicted in Figure 5a. Favorably, our Zn/ZnO battery could harvest a maximum energy density of 422 W h kg^−1^ at a power density of 150 W kg^−1^. This energy output was much higher than the documented traditionally applicable aqueous zinc-ion supercapacitors [39], Zn/NiO batteries [40], Al-manganese batteries [41], Ni_3_S_2_/Ni batteries [42], Fe-I_2_ batteries [43], Zn-graphite batteries [44], Zn/V_2_O_5_ batteries [45], Zn/ppy batteries [46], LiFePO_4_/Fe batteries [47], and aqueous magnesium-ion batteries [48]. Our Zn/ZnO battery exhibited a high voltage with an enriched charge/discharge plateaus and high energy and power outputs, demonstrating the efficacy of the present Zn/ZnO battery for mass-scale applications.

The superior electrochemical properties of the present coin-cell Zn/ZnO battery motivated us to translate this success into pouch Zn/ZnO cells considering the importance of the battery configuration of the latter in a grid-scale energy storage application. The pouch Zn/ZnO cells were constructed by sandwiching the glass-fiber separator and ZMS electrolyte between the ZnO cathode and the metallic Zn foil, and it was then used to power an electronic watch. The digital numbers on the screen were observed with the help of a single soft-packed pouch Zn/ZnO cell (Figure 5b). To test the electrochemical endurance of a single Zn/ZnO pouch cell, the rate performance evolution at various current rates was performed, and the corresponding charge/discharge pattern exhibited a similar shape as that of the coil-cell-based Zn/ZnO construction with an average discharge voltage of 1.5 V (Figure S12b).

Furthermore, the superior electrochemical capacities of 214, 201, 194, 177, 151, 141, and 120 mAh g^−1^ at current rates of 150, 300, 500, 750, 1000, 1500, and 2000 mA g^−1^, respectively, were achieved (Figure 5c). The superior rate performance with high mass loading was comparable with that of the coin-cell-based Zn/ZnO assembly. More importantly, the integrated Zn/ZnO pouch cells were designed via the connection of multiple cells in series (Figure 5d). It was encouraging that the charge/discharge profiles of the cells connected in series depicted identical electrochemical features and caused a consequent stepwise rise in the output potential from 1.5 V for a single cell to 3 V for two cells and 4.5 V for three cells, which was indicative of phenomenal enactment repetition. Two serially connected Zn/ZnO pouch cells maintained a high boosted-up voltage (after the fifth charge) of 3.76 V and lit up a single LED (Figure 5e–f) as well as 1 m long 3–6 V serial light-emitting diodes (LEDs) for a vitally long time (Figure 5g), substantiating the tremendous capability of our integrated Zn/ZnO pouch batteries for practical applications. Accordingly, our soft-packed Zn/ZnO battery manifested the benefits of being eco-friendly, cheap, and adaptable in addition to having the safety aspects of a modern battery. Furthermore, it could retain high energy and power densities, rendering them applicable for various appliances in an uninterrupted manner.

The proposed Zn/ZnO battery had both its advantages and shortcomings. From our research findings, it was verified that the electrolyte was the prime capacity donor. However, the aqueous Zn-based systems with Mn-containing electrolytes involved complicated parasitic reactions, and it was extremely difficult to precisely locate the participation of Mn^2+^ ions in the capacity contribution [49]. Similarly, it was very challenging to precisely locate the participation of ZnO in the capacity contribution of the Zn/ZnO system (ZnO ↔ ZBS ↔ MnO_2_). In a recent study, the conversion reaction between ZnO → ZBS ↔ Zn_x_MnO(OH)_2_ was verified using higher-end characterization techniques [50]. Hence, to understand the proposed reaction mechanism in this study, additional experimental data and advanced characterization methods were essential.

## 3. Materials and Methods

### 3.1. Synthesis of ZnO Microspheres

ZnO microspheres were synthesized according to a previous report [29]. More specifically, zinc acetate·dihydrate (1 g) was dissolved in 10 mL of deionized water. After stirring the solution for a few minutes, it was mixed with a 60 mL glycerol solution to obtain a homogenous solution, which was subsequently transferred to an airtight Teflon-lined autoclave maintained at 200 °C for 12 h in an electric oven after which the autoclave was allowed to cool naturally. The resulting precipitate was collected, washed multiple times with distilled water and ethanol, and then dried in an oven at 80 °C for 8 h before characterization.

### 3.2. Structural and Morphological Characterization

A Shimadzu X-ray diffractometer was used for powder X-ray diffraction (PXRD, Cu Kα radiation, λ = 1.5406 Å) measurements. Field-emission scanning electron microscopy (FE-SEM, Hitachi S-4700) with an energy-dispersive X-ray spectroscopy (EDS) detector was employed to characterize the sample surface morphologies. Field-emission transmission electron microscopy (FE-TEM, Philips Tecnai, F20, 200 kV, Korea Basic Science Institute, Chonnam National University, Amsterdam, Netherlands) with selected-area electron diffraction (SAED) was used to study the structural morphologies and lattice fringes. The surface oxidation states of the electrode samples were determined through X-ray photoelectron spectroscopy (XPS, Thermo VG Scientific, MultiLab 2000, Chonnam Center for Research Facilities, Al Kα X-ray source, Waltham, MA, USA). The spectrometer was calibrated with respect to C 1s at 284.8 eV.

### 3.3. Electrochemical Characterization

For the electrochemical measurements, the electrode was prepared using the doctor blade method. Homogeneous slurries were prepared by mixing 70%, 15%, or 15% active material; Super P conductive carbon; and a polyacrylic acid binder in N-methyl-2-pyrrolidone. Then, the slurry was coated uniformly onto a stainless-steel foil, which functioned as a current collector, and it was subsequently dried overnight under vacuum at 120 °C before being pressed between stainless-steel twin rollers (maintained at 120 °C). The foil was then punched into round discs. Active mass loadings were in the range of 1.4–2.5 mg. CR 2032 coin cells were constructed using the ZnO electrodes with zinc metal foil as an anode and a combination of 1 M ZnSO_4_ and 0.2 M MnSO_4_ as an electrolyte. For the pouch cells, the mass loading was found to be in the range of 7–9 mg. Additionally, 1 M ZnSO_4_ (ZS), 1 M MgSO_4_ + 0.2 M MnSO_4_ (MMS), Na_2_SO_4_ + 0.2 M MnSO_4_ (NMS), and 0.2 M MnSO_4_ (MS) were also used as electrolytes. A glass-fiber separator was soaked in the electrolyte (a persistent volume of 150 µL) and was subsequently pressed between the prepared ZnO cathode and anode under ambient conditions. The electrochemical charges and discharges were successively measured using a battery tester (Nagano, BTS-2004H) at various current densities in the working voltage range of 1.9–0.6 V vs. Zn^2+^/Zn. A potentiostat model workstation (Autolab, PGSTAT302N) was adopted for cyclic voltammetry (CV) scans. The theoretical capacity calculation of the Zn/ZnO system is provided in Supplementary Note 2.

### 3.4. Specific Energy and Specific Power Calculation

The specific energy was calculated as E (Wh kg^−1^) = specific capacity × potential (average working potential).

The specific power was calculated as P (W kg^−1^) = I × V/2m, where I is the applied current (A), V is the average working potential (V), and m is the active mass of the cathode side [13].

## 4. Conclusions

In the quest to develop safe, low-cost, grid-scale ESSs, aqueous Zn/ZnO-based batteries operated by reversible metal deposition/dissolution in mildly acidic sulfate electrolytes are excellent candidates. When tested as the cathode, ZnO microspheres easily boosted reversible MORs/MRRs, resulting in a superior rate performance (76 mAh g^−1^ at 5000 mA g^−1^) and superior cycling stability (i.e., a reversible capacity of ≈68 mAh g^−1^ after 1000 cycles at a high current density of 3000 mA g^−1^). The reason for the enhanced performance in the Zn/ZnO battery was attributed to the unique reactions of ZnO. The ZnO electrode played a sacrificial role via the irreversible transformation into ZBS early in initial charge cycling. Then, the transformed ZBS phase underwent reversible self-disintegration, activating a MOR to deposit MnO_2_ on the electrode surface after complete initial charging. The MRR related to the dissolution of solid MnO_2_ into aqueous Mn^2+^ ions drove the discharge cycling. Thus, the reversible MOR/MRR related to the self-disintegration/regeneration of ZBS, respectively, caused the Zn/ZnO battery to achieve high energy densities capable of suiting the needs of large-scale grid applications. Therefore, the present study motivated the investigation of other various prospective materials, including metal oxides, sulfides, or phosphates that can demonstrate facile MORs/MRRs for prospective electrode applications in high-energy rechargeable aqueous batteries.

## Data Availability

Not applicable.

## Supplementary Materials

The following supporting information can be downloaded at https://www.mdpi.com/article/10.3390/molecules27248664/s1; Table S1: Rietveld refinement results of the ZnO samples using XRD data; Figure S1: XRD patterns of ZnO powder immersed in ZMS and ZS solutions for 1 h; Figure S2: (a) FTIR spectra of the ZnO microsphere before and after immersion in ZS and ZMS solutions for 1 h, respectively. (b) Zn 2p and (c) S 2p XPS profiles of the ZnO microsphere before and after immersion in ZS and ZMS solutions for 1 h; Figure S3: Survey spectrum of ZnO powder immersed in ZS and ZMS solutions for 1 h along with pristine ZnO powder; Figure S4: Selected charge/discharge patterns of ZnO microspheres cycled at different rates in ZMS electrolyte; Figure S5: XRD patterns of ZnO electrode in ZMS electrolyte for 1h and 24 h along with pristine ZnO electrode; Figure S6: FEM images of ZnO electrode in ZMS electrolyte for 1 h (a–b) and (c–d) 24 h; Figure S7: Ex situ XRD analysis at different depths of charge/discharge voltage in MS electrolyte; Figure S8: Ex situ TEM image of ZnO samples at 100 mA g^−1^ current density in ZMS electrolyte, (a–c) microflowers, (d–e) hierarchal microrods, and (g–i) microfibers; Figure S9: Digital photographs of postmortem samples after initial (a) charge and (b) discharge; Figure S10: Ex situ SEM analysis of the glass fibers after the 1st charge process in ZMS electrolyte; Figure S11: Cycle patterns of ZnO microspheres cycled at 100 mA g^−1^ in (a) NMS and (b) MMS electrolytes; Figure S12: (a) Ex situ XRD patterns of ZnO microspheres after the 100th discharge cycle in MMS and NMS electrolytes and (b) selected charge/discharge patterns of Zn/ZnO pouch battery cycled at different rates in ZMS electrolyte. Reference [51] is cited in the supplementary materials.
