# Supplementary material for: Aqueous Rechargeable Zn/ZnO Battery Based on Deposition/Dissolution Chemistry
Source: Molecules. 2022 Dec 7;27(24):8664. doi: 10.3390/molecules27248664 (PMC9786327; doi:10.3390/molecules27248664)
Supplement: Supplementary file 1 [file molecules-27-08664-s001.zip › molecules-2006706-supplementary.pdf]

Supplementary Information

Aqueous Rechargeable Zn/ZnO Battery Based on Deposition/Dissolution Chemistry

Vaiyapuri Soundharrajan[1,†], Jun Lee[1,†], Seokhun Kim[1], Dimas Yunianto Putro[1], Seulgi Lee[1], Balaji Sambandam[1], Vinod Mathew[1], Kumaresan Sakthiabirami[2], Jang-Yeon Hwang[1,] and Jaekook Kim[1,*]*

Supplementary Note 1

The powder XRD pattern was fitted using the FullProf software for high accuracy.

Table S1. Rietveld refinement results of the ZnO samples using XRD data.

**ZnO**						
**Space group**			*P63m c (No. 186)*			
***a* (Å), *c* (Å), V (Å)**			3.2499, 5.2110, 47.6638			
**atom**	**site**	**Wyckoff positions**			**occupancy[a]**	***B* ($Å^2$)**
Zn[b]	2b	0.3333	0.6666	0	1[c]	1.319
O[b]	2b	0.3333	0.6666	0.3693(1)	1[c]	1.367
Reliability factors			R_p = 13.2 %, R_{wp} = 18.6 %, R_{exp} = 9.66 %, χ^2 =3.70			

[a] The site occupation numbers are in %; [b] the occupancies were calculated using the following constraints: $(Occ)Zn_{2b} = (Occ)O_{2b}$; [c] fixed parameter.

Supplementary Note 2

Theoretical capacity calculation:

The theoretical capacity can be calculated from the number of moles of electrons involved in the reaction according to Faraday's law [51]:

Theoretical capacity, $T_C = 26800\ n/M$ -- (1)

where n= the number of moles of electrons involved in the reaction and M= the molar mass of the active material.

The deposition/dissolution of electrolytic MnO_2 was associated with the formation/disappearance of ZBS, which was sourced from the ZnO cathode. However, it was complicated to estimate the exact quantity of ZBS formed from ZnO and its involvement in MnO_2 formation. Hence, we still used the mass of ZnO to interpret the specific capacity and energy density calculation. For the Zn/MnO_2 system, the theoretical capacity was calculated to be 616.58 mAh/g if two electrons were involved. Similarly, for the Zn/ZnO system, we assumed that two electrons were involved during the electrochemical process. The theoretical capacity was found to be 658.63 mAh/g, which was close to the Zn/MnO_2 system.

Supplementary Note 3

Chemical reaction of commercial ZnO powder in sulfate-based aqueous solutions:

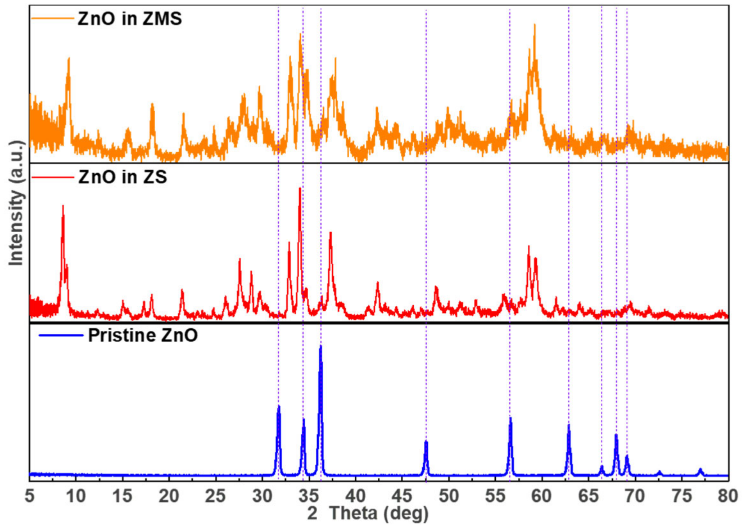

Figure S1. XRD patterns of ZnO powder immersed in ZMS and ZS solutions for 1 h.

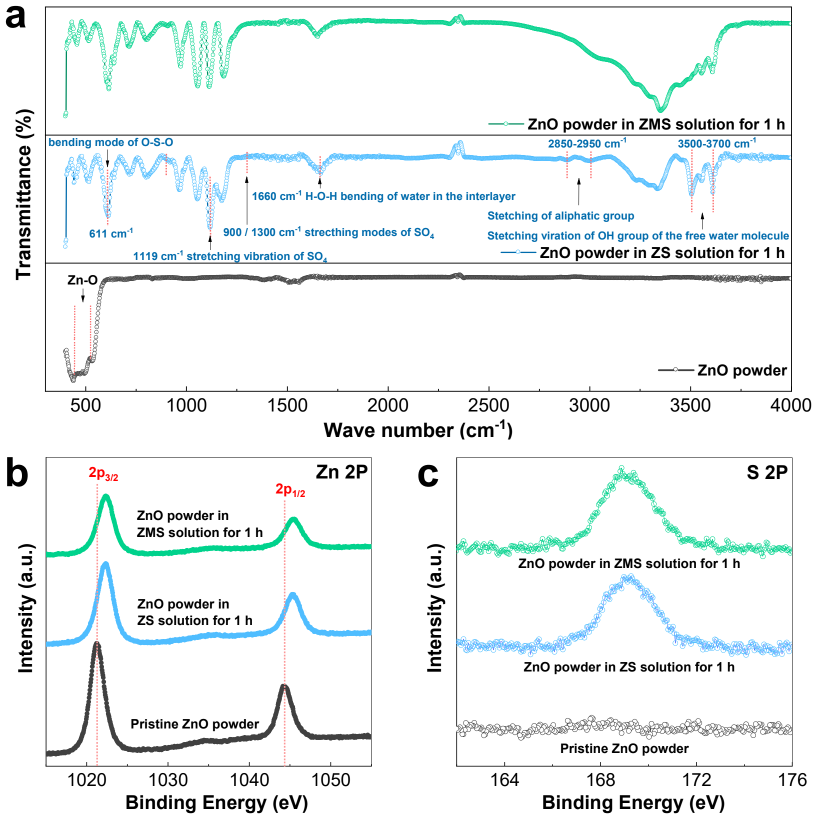

Figure S2. (**a**) FTIR spectra of the ZnO microsphere before and after immersion in the ZS and ZMS solutions for 1 h, respectively. (**b**) Zn 2p and (**c**) S 2p XPS profiles of the ZnO microsphere before and after immersion in the ZS and ZMS solutions for 1 h.

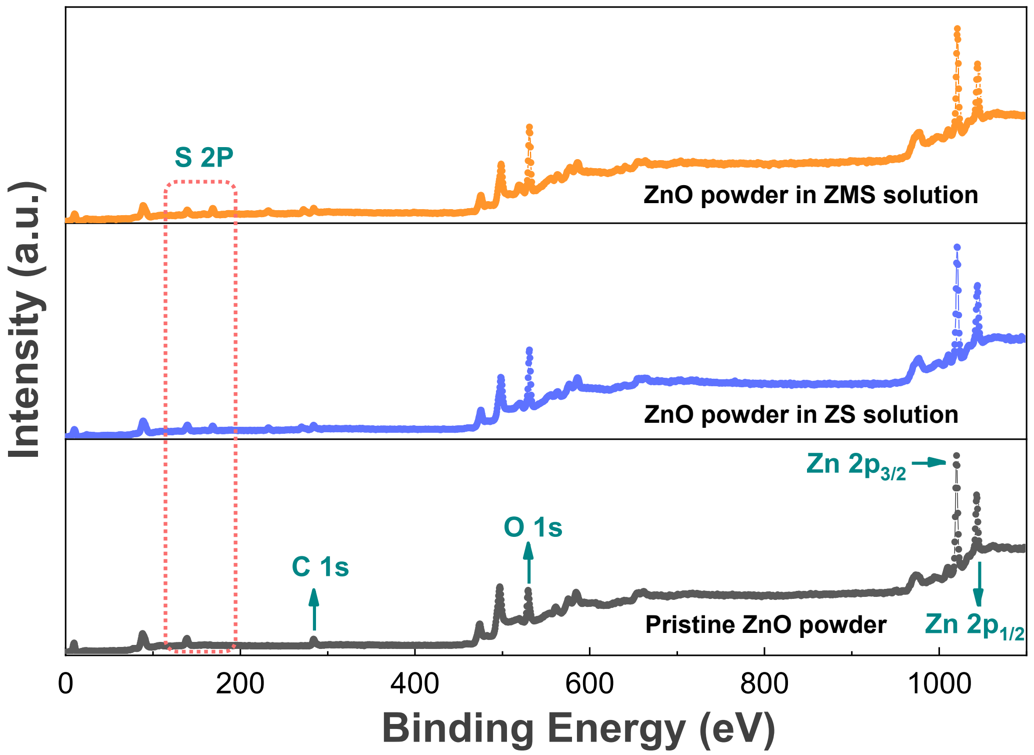

Figure S3. Survey spectrum of ZnO powder immersed in ZS and ZMS solutions for 1 h along with pristine ZnO powder.

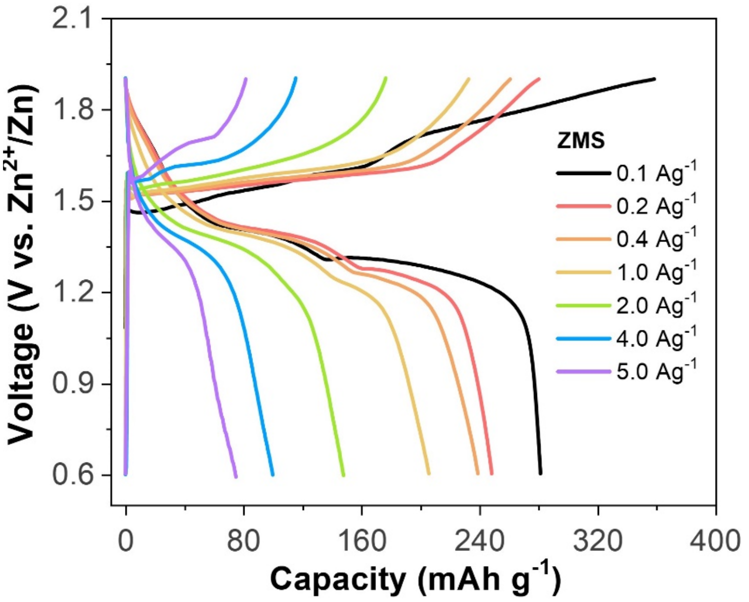

Figure S4. Selected charge/discharge patterns of ZnO microspheres cycled at different rates in ZMS electrolyte.

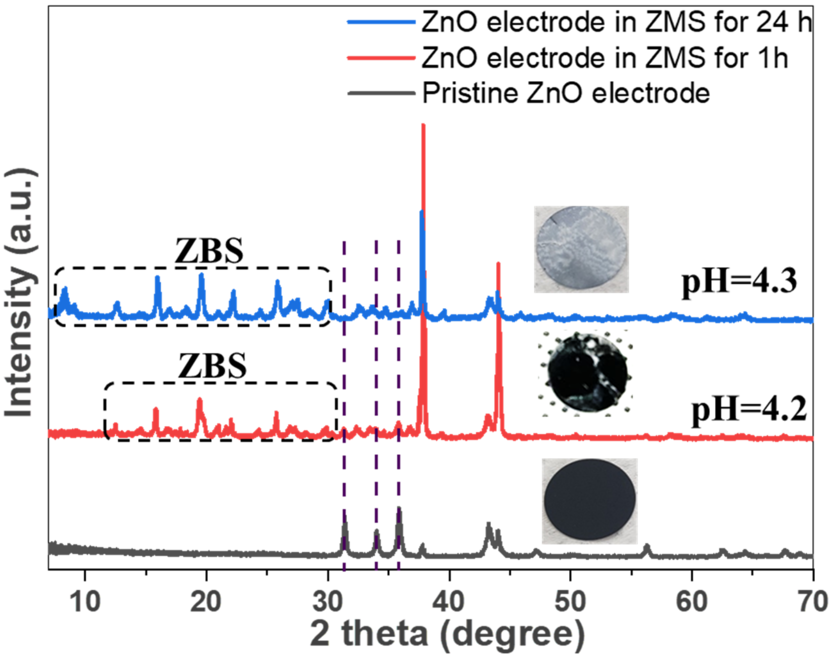

Figure S5. XRD patterns of ZnO electrode in ZMS electrolyte for 1h and 24 h along with pristine ZnO electrode.

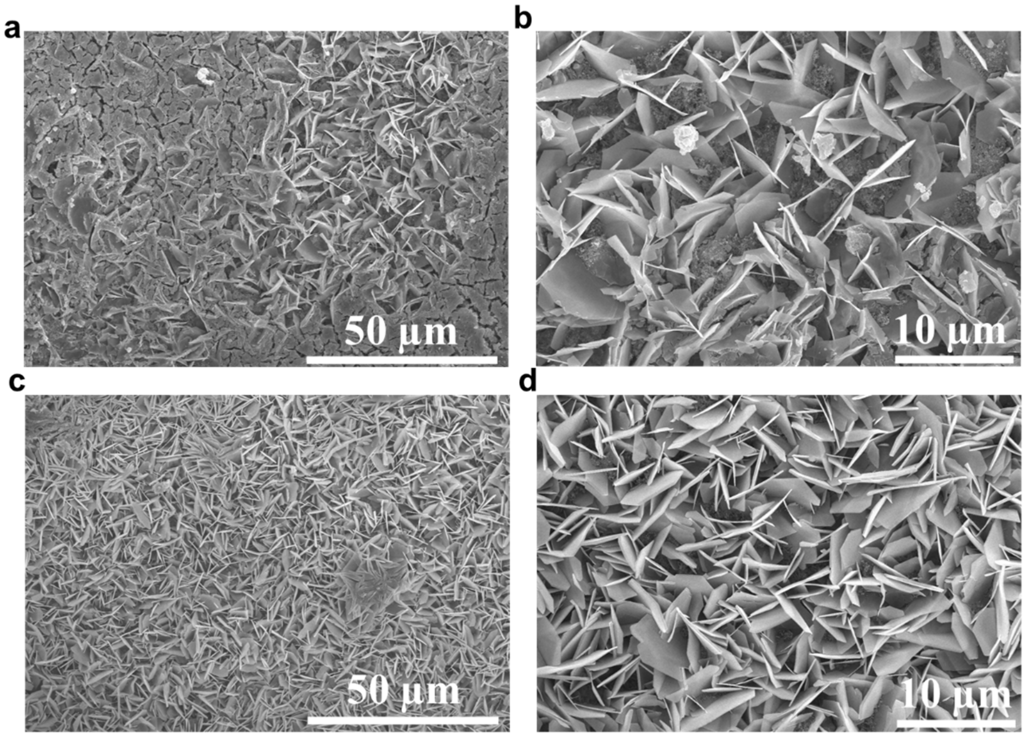

Figure S6. FEM images of ZnO electrode in ZMS electrolyte for 1h (**a,b**) and (**c,d**) 24 h.

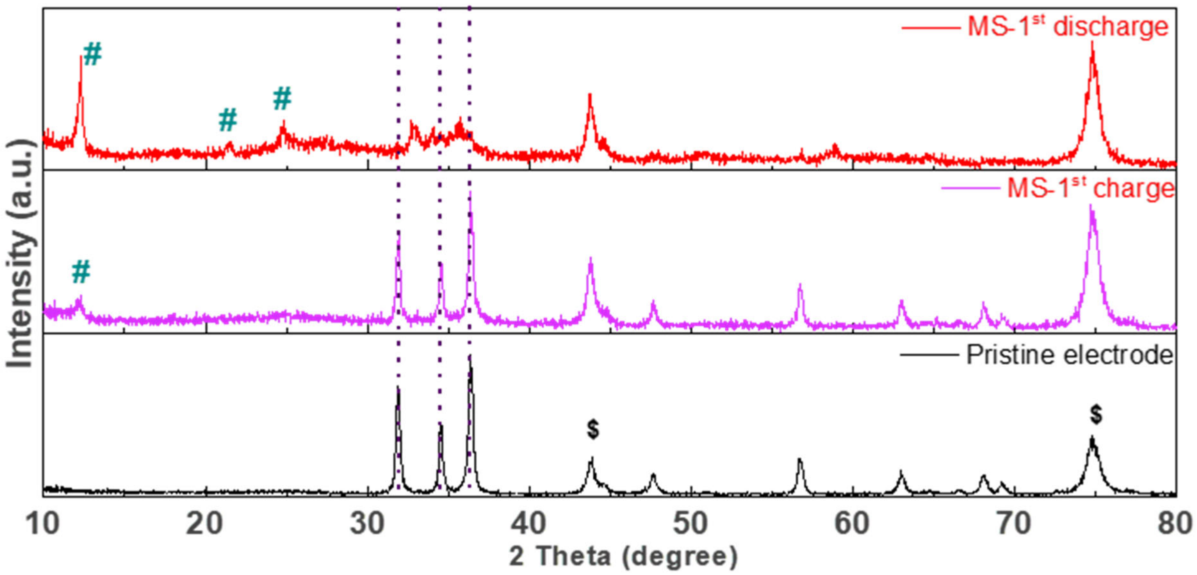

Figure S7. Ex situ XRD analysis at different depths of charge/discharge voltage in MS electrolyte.

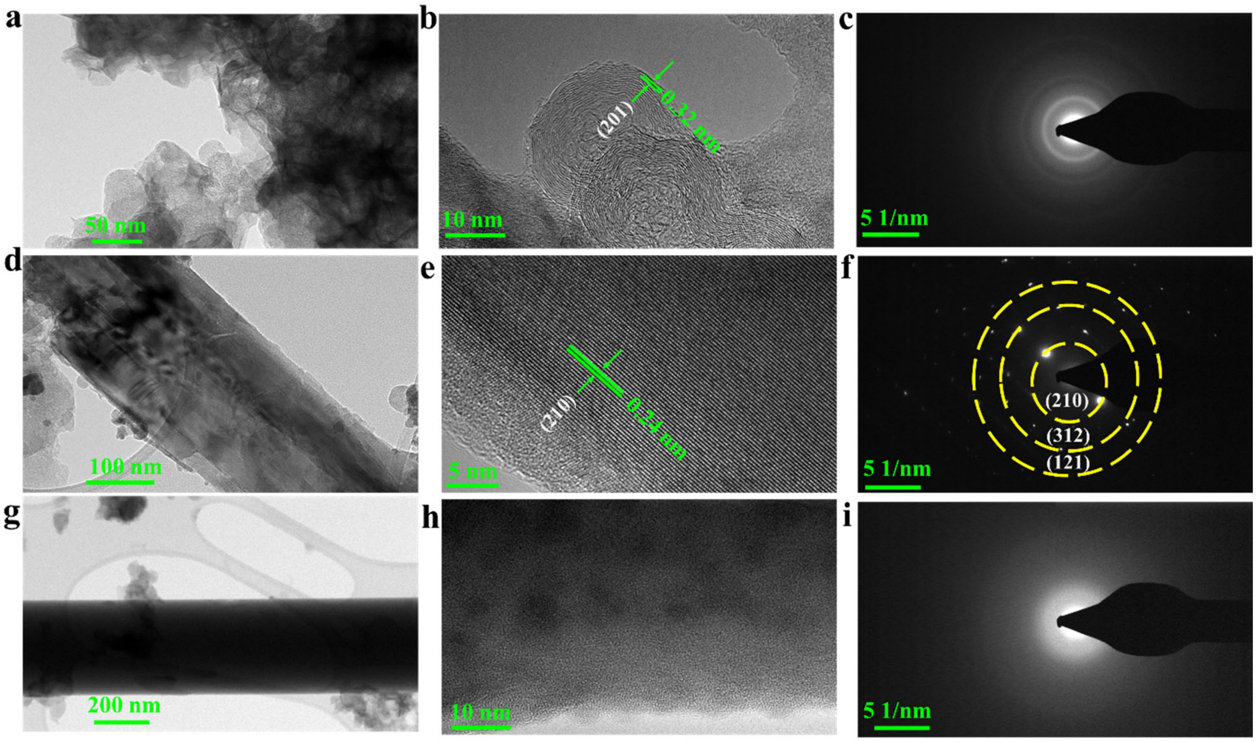

Figure S8. *Ex situ* TEM image of ZnO samples at 100 mA g^{-1} current density in ZMS electrolyte, (**a–c**) microflowers, (**d,e**) hierarchal microrods, and (**g–i**) microfibers.

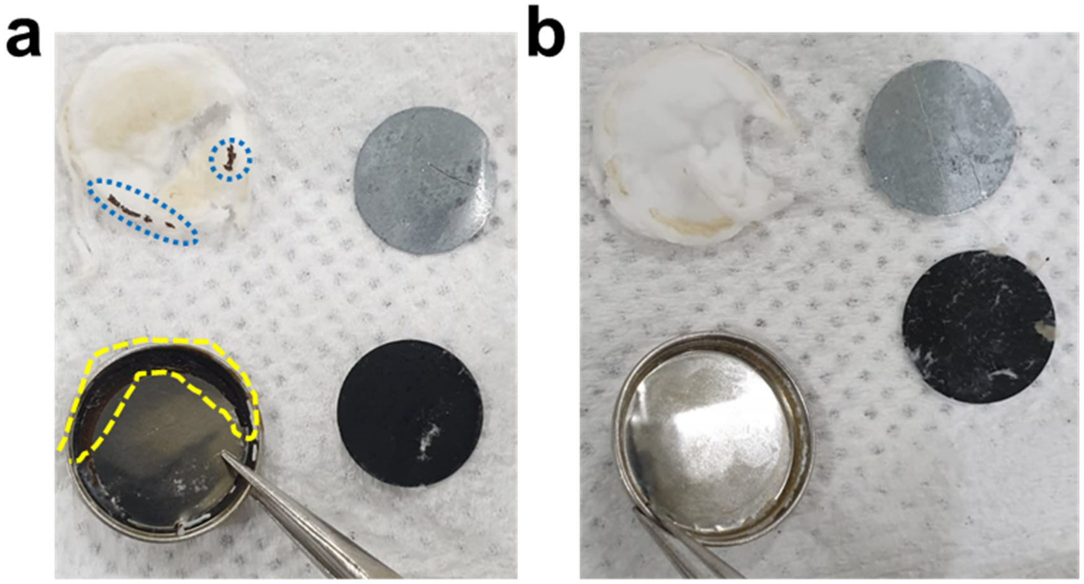

Figure S9. Digital photographs of postmortem samples after initial (**a**) charge and (**b**) discharge.

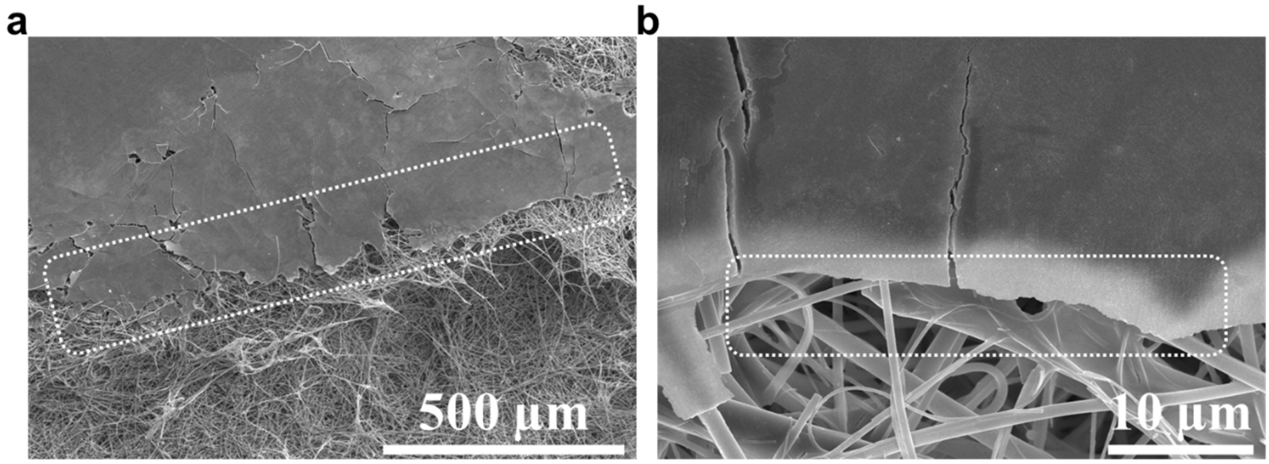

Figure S10. Ex situ SEM analysis of the glass fibers after the 1st charge process in ZMS electrolyte.

Supplementary Note 4

Zn/ZnO battery diversity and feasibility for likely practical applications:

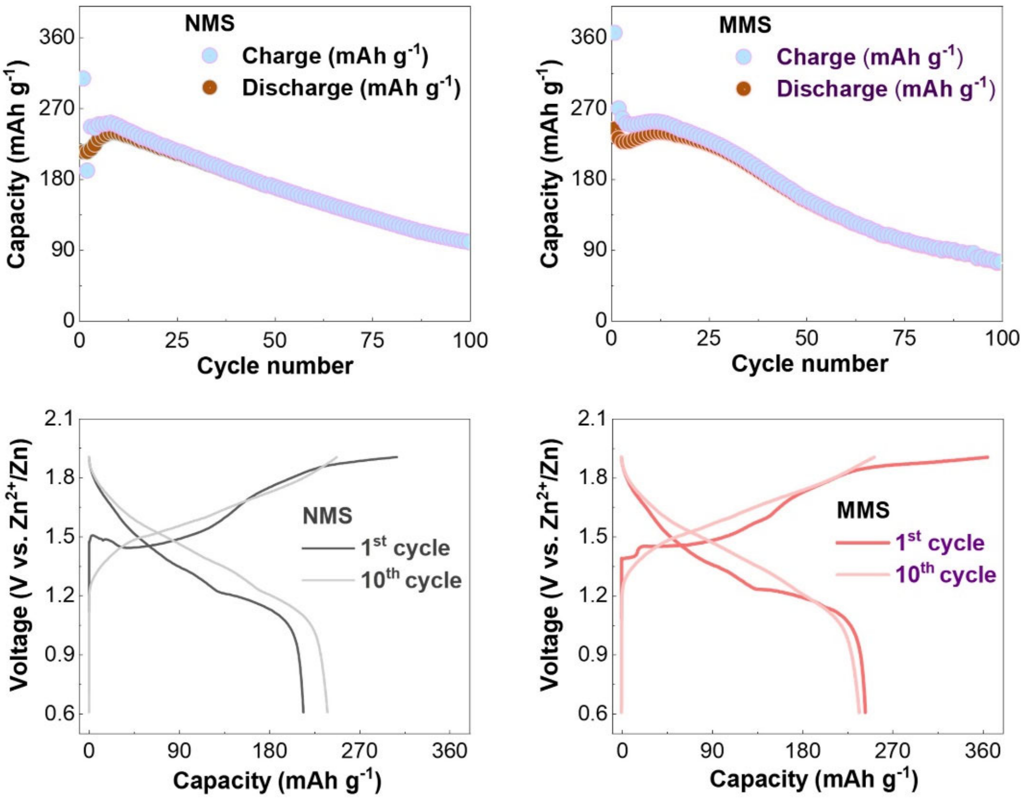

Figure S11. Cycle patterns of ZnO microspheres cycled at 100 mA g^{-1} in (**a**) NMS and (**b**) MMS electrolytes.

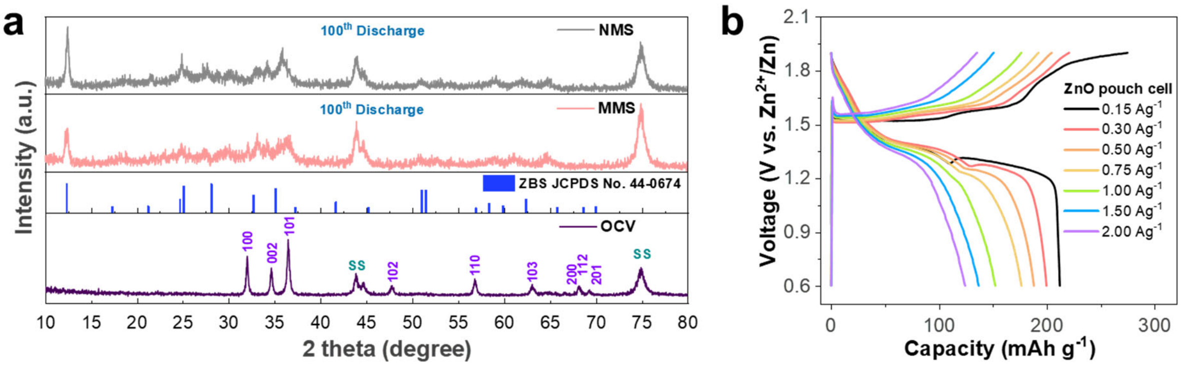

Figure S12. (**a**) *Ex situ* XRD patterns of ZnO microspheres after the 100th discharge cycle in MMS and NMS electrolytes and (**b**) selected charge/discharge patterns of Zn/ZnO pouch battery cycled at different rates in ZMS electrolyte.

Furthermore, to demonstrate the diversity of the proposed reversible MnO_2-deposition/dissolution-induced charge storage mechanism, we designed Zn/ZnO

batteries with diverse electrolyte solutions containing 1 M Na_2SO_4 or 1 M $MgSO_4$ salts. To both the aforementioned electrolytes, $MnSO_4$ additive was added to form NMS (1 M Na_2SO_4 + 0.2 M $MnSO_4$) and MMS (1 M Mg_2SO_4 + 0.2 M $MnSO_4$) electrolytes to promote *in situ* MnO_2 deposition/dissolution during cycling. The Zn/ZnO batteries prepared with the additive-containing electrolytes were galvanostatically tested at 100 mA g^{-1}, and the corresponding electrochemical outputs are provided in **Figure S6**. It is fascinating that the ZnO charge/discharge curves of all the electrolytes showed patterns identical to those of ZMS. Interestingly, the ZnO electrode delivered high initial manganese deposition/dissolution efficiencies of 69% and 66% in the NMS and MMS electrolytes, respectively. Moreover, the ZnO electrode showed poor cycling stability in both electrolytes for 100 consecutive cycles. Specifically, after delivering the high discharge capacities of 215 mAh g^{-1} and 244 mAh g^{-1}, the ZnO electrode delivered the stable reversible capacities of 99 mAh g^{-1} and 73 mAh g^{-1} in the NMS and MMS electrolytes, respectively. This clearly showed that manganese deposition/dissolution was reversible irrespective of the charge carriers. Furthermore, we conducted *ex situ* XRD evolutions for the Zn/ZnO battery in NMS and MMS electrolytes to confirm the reversibility of the ZBS formation, which was responsible for the MnO_2 deposition/dissolution mechanism. Even after the 100th discharge cycle (≈ 0.6 V DOD) in both electrolytes, the *ex situ* XRD patterns of the electrodes still exhibited ZBS peaks (**Figure S7**), confirming the stated reaction mechanism for Zn/ZnO cells containing sulfate-based electrolytes.
